# Incidence and Antiseizure Medications of Post-stroke Epilepsy in Umbria: A Population-Based Study Using Healthcare Administrative Databases

**DOI:** 10.3389/fneur.2021.800524

**Published:** 2022-01-12

**Authors:** Cinzia Costa, Elena Nardi Cesarini, Paolo Eusebi, David Franchini, Paola Casucci, Marcello De Giorgi, Carmen Calvello, Michele Romoli, Lucilla Parnetti, Paolo Calabresi

**Affiliations:** ^1^Neurology Clinic, Department of Medicine and Surgery, Santa Maria della (S.M.) Misericordia Hospital, University of Perugia, Perugia, Italy; ^2^UOC Neurologia, Ospedale di Senigallia, Senigallia, Italy; ^3^Health ICT Service, Regional Health Authority of Umbria, Perugia, Italy; ^4^Neurology and Stroke Unit, “Maurizio Bufalini” Hospital, Cesena, Italy; ^5^Neurologia, Dipartimento Neuroscienze, Università Cattolica del Sacro Cuore, Rome, Italy; ^6^Neurologia, Fondazione Policlinico Universitario “A. Gemelli” IRCCS, Rome, Italy

**Keywords:** incidence, epilepsy, stroke, antiseizure medications, administrative databases

## Abstract

**Introduction:** Post-stroke epilepsy (PSE) requires long-term treatment with antiseizure medications (ASMs). However, epidemiology of PSE and long-term compliance with ASM in this population are still unclear. Here we report, through population-level healthcare administrative data, incidence, risk factors, ASM choice, and ASM switch over long-term follow-up.

**Materials and Methods:** This is a population-based retrospective study using Umbria healthcare administrative database. Population consisted of all patients with acute stroke, either ischaemic or hemorrhagic, between 2013 and 2018. ICD-9-CM codes were implemented to identify people with stroke, while PSE was adjudicated according to previously validated algorithm, such as EEG and ≥1 ASM 7 days after stroke.

**Results:** Overall, among 11,093 incident cases of acute stroke (75.9% ischemic), 275 subjects presented PSE, for a cumulative incidence of 2.5%. Patients with PSE were younger (64 vs. 76 years), more frequently presented with hemorrhagic stroke, and had longer hospital stay (15.5 vs. 11.2 days) compared with patients without PSE. Multivariable Cox proportional hazards models confirmed that PSE associated with hemorrhagic stroke, younger age, and longer duration of hospital stay. Levetiracetam was the most prescribed ASM (55.3%), followed by valproate and oxcarbazepine. Almost 30% of patients prescribed with these ASMs switched treatment during follow-up, mostly toward non-enzyme-inducing ASMs. About 12% of patients was prescribed ASM polytherapy over follow-up.

**Conclusions:** Post-stroke epilepsy is associated with hemorrhagic stroke, younger age, and longer hospital stay. First ASM is switched every one in three patients, suggesting the need for treatment tailoring in line with secondary prevention.

## Introduction

Stroke is the third leading cause of death and a major cause of disability in developed countries, with an estimated 12 million cases annually (1). Stroke survivors have an increased risk of seizures, with stroke being the most common cause of acquired epilepsy in adults, accounting up to 70% of cases (2–4). People with stroke can experience both early and late seizures. Early seizures, occurring <7 days after stroke, and late seizures (>7 days) can both happen in people with stroke. The former, occurring within the first 7 days from stroke symptoms, is thought to derive from local cytotoxic neurotransmitters release, able to induce neuronal hyperexcitability in the penumbra area in the acute phase. The incidence of early seizures after stroke reaches 6%, and seems higher in people with intracerebral (ICH) or subarachnoid hemorrhage (SAH) (5, 6). Despite early seizures can be the harbinger of structural epilepsy, late seizures develop in fewer stroke survivors (2). However, as for the International League Against Epilepsy definition, a single late seizure after stroke qualifies as structural epilepsy (post-stroke epilepsy: PSE) due to the high (>60%) risk of recurrence within the next 10 years (7, 8). The latent period between stroke onset and the first late seizure suggests that the late seizures and PSE reflect a structural remodeling of neural networks following cerebrovascular injury rather than a simple direct stress mechanism (3, 9). PSE associates carry higher mortality and negatively impact the quality of life (10, 11). Moreover, antiseizure medications (ASMs) can interact with antithrombotic, further increasing the complexity of secondary prevention strategies.

Despite several studies addressed prevalence and long-term impact of PSE, few were derived from population-level data, and none addressed the long-term compliance of first-choice ASM.

This population-based retrospective study aimed at defining the incidence of PSE and its treatment over time in real-world setting using the healthcare administrative data.

## Materials and Methods

### Study Design

This is an observational, population-based retrospective study based on linkage from the regional healthcare administrative databases. The databases cover the healthcare utilization of 900,000 people in Umbria Region, in central Italy. The database contains anonymized, individual-level data regarding hospitalization, diagnostic work-up, in/out of hospital visit or diagnosis, and medication prescriptions.

The population study consisted of all patients with a hospitalization due to acute stroke in Umbria from January 01, 2013 to December 31, 2018. To allow a follow-up time of at least 12 months after first hospitalization, all patients with acute stroke were followed-up to December 31, 2019. To limit bias related to previous stroke, an observation period of 1 year before January 01, 2013 (January 01, 2012 to December 31, 2012) was defined to exclude patients with previous stroke or seizures.

### Case Ascertainment

Patients with a first hospital discharge for stroke, between 2013 and 2018, were identified in the administrative databases using the following groups of ICD-9-CM codes located in primary position: 433 × 1, 434 (excluding 434 × 0), 436 for ischemic stroke; 430 and 431 for hemorrhagic stroke (subarachnoid hemorrhage, SAH, and intracerebral hemorrhage).

The records collected during the years 2012–2019 were linked to ascertain cases of epilepsy according to previously reported algorithm (12), which adjudicated epilepsy with at least one EEG and one or more ASM, either as monotherapy or combination. The previous algorithm was tested in the Italian healthcare administrative databases with good practice (GP) diagnosis as gold standard, with an accuracy of 94.8% (85.9% sensitivity and 99.8% specificity) (12).

For the diagnosis of PSE, we included all people diagnosed with stroke that underwent EEG and took one or more ASM (ATC-code N03A) after 7 days from the stroke onset. We collected the following ASM: carbamazepine (CBZ), phenytoin (PHT), phenobarbital (PB), primidone (PRM), barbexaclone (BSC), clonazepam (CLZ), ethosuximide (ESM), valproate (VPA), valpromide (VPM), clobazam (CLB), vigabatrin (VGB), felbamate (FLB), tiagabine (TGB), pregabalin (PRG), oxcarbazepine (OXC), gabapentin (GBP), topiramate (TPM), levetiracetam (LEV), zonisamide (ZNS), lamotrigine (LTG), lacosamide (LCM), perampanel (PER), brivaracetam (BRV), and eslicarbazepine acetate (ESL).

Treatment start was defined as the first dispensation date of an ASM. Treatment continuation was defined as 3 or more dispensations per year, while treatment end was adjudicated when 12 or more months elapsed from the last ASM dispensation. Patients with dispensations of a second ASM within 1 week of the first were categorized as combination. Hypertension and diabetes were identified as an occurrence of each diagnosis or use of specific medications through administrative healthcare regional data. The Regional ethical review board approved the study (no 3420/19).

### Statistical Analysis

All data were checked for their validity based on pre-defined algorithms. Incidence rates were calculated along with their 95% Confidence Interval (CI). For categorical (including dichotomous) variables, frequencies and percentages were reported. Statistical comparisons to assess differences in the distribution of categorical variables between groups were conducted using chi-square tests. Continuous variables were summarized using mean and Standard Deviations (SD). *T*-tests were used for comparing the distributions of continuous variables. Cox proportional hazards models were carried out for assessing the role of demographics, comorbidities, and characteristics of index hospitalization (type of stroke and length of stay as surrogate measure of stroke severity) in the prediction of the onset of PSE. Results of Cox proportional hazards models are presented as hazard ratios (*HR*s) with 95% *CI*. Significance level of 5% was assumed for all the analyses. Statistical analyses were performed using R software version 4.0. This study was conducted in accordance with known guidelines for observational studies (STROBE and ISPOR) (13).

## Results

### Cohort Characteristics

During the study period (2013–2018), 11,093 incident cases of acute stroke were identified. Most of our cerebrovascular events were ischemic strokes (*n* = 8,428, 76.0%; intracerebral hemorrhages *n* = 2,184, 19.7%; subarachnoid hemorrhages *n* = 481, 4.3%). During hospital stay 1,368 patients with stroke died, and were therefore excluded, leaving 9,725 in the final cohort. The incidence rate of stroke diagnosis in Umbria during 2013–2018 was of 184–229 per 100,000 persons-year. Overall, 9,725 stroke survivors were followed-up until 2019, of which 275 (2.8%) developed PSE. The patients with PSE were younger (64.0 ± 16.0 vs. 75.7 ± 13.0 years, *p* < 0.001) and had longer duration of hospital stay (15.5 ± 12.9 vs. 11.2 ± 9.2 days, *p* < 0.001) compared with non-PSE subjects (Table 1). No differences in gender and comorbidities, such as hypertension and diabetes, were detected. Hemorrhagic stroke and SAH were more frequent among people developing PSE vs. non-PSE group (intracerebral hemorrhagic 33.4 vs. 15.3% and SAH 12.4 vs. 3.9%).

**Table 1:** Demographic characteristics of people with and without PSE.

	**Patients with stroke (2013–2018)**		***p*-value**
	**PSE (*n* = 275)**	**No PSE (*n* = 9,450)**	
**Gender (** ***n*** **, %)**	
Male	157 (57.1)	4,853 (51.4)	0.070
Female	118 (42.9)	4,597 (48.6)	
**Age, years**	64.0 ± 16.0	75.7 ± 13.0	**<0.001**
**Length of stay, days**	15.5 ± 12.9	11.2 ± 9.2	**<0.001**
**Stroke type (** ***n*** **, %)**	
Ischaemic	149 (54.2)	7,638 (80.8)	**<0.001**
Intracerebral haemorrhagic	92 (33.4)	1,448 (15.3)	
Subarachoid haemorrhagic	34 (12.4)	364 (3.9)	
**Diabetes (** ***n*** **, %)**	
No	209 (76.0)	6,784 (71.6)	0.143
Yes	66 (24.0)	2,666 (28.4)	
**Hypertension (** ***n*** **, %)**	
No	71 (25.8)	2,062 (21.8)	0.132
Yes	204 (74.2)	7,388 (78.2)	

*p ≤ 0.001*.

### Incidence Rates

The overall cumulative incidence rate of PSE during the first year after stroke was 2.3% (95% *CI* = 2.0–2.7%), with 7.5% (95% *CI* = 5.2–10.5%) in SAH, 5.4% (95% *CI* = 4.3–6.6%) in intracerebral hemorrhage, and 1.5% (95% *CI* = 1.2–1.8%) in ischemic stroke. The overall cumulative incidence rate at 4 years after stroke was 3.1% (95% *CI* = 2.7–3.5%), with 9.3% (95% *CI* = 6.6–12.6%) in SAH, 6.3% (95% *CI* = 5.1–7.7%) in intracerebral hemorrhage, and 2.1% (95% CI = 1.8–2.5%) in ischemic stroke.

### Risk Factors

Univariate Cox regression confirmed the association of PSE onset with hemorrhagic stroke, younger age, and longer hospital stay. All the significant factors at univariate analysis entered in the multivariable Cox regression model (c-index = 0.71), which showed that onset of PSE was associated with hemorrhagic stroke (*HR* for intracerebral hemorrhage = 2.76, 95% *CI* = 1.85–4.12; *HR* for SAH=2.84, 95% CI = 2.17–3.71), younger age (*HR* = 2.64, 95% *CI* = 2.06–3.38), and longer duration of hospital stay (*HR* = 1.11, 95% *CI* = 1.05–1.17) (Table 2). No other available variable was associated with PSE.

**Table 2:** Multivariate cox regression modeling of independent predictors of PSE.

	**Hazard ratio (95% CI)**	***p*-value**
**Gender**	
Male	–	0.482
Female	0.87 (0.69–1.11)	
**Age**	
≥65 years	–	**<0.001**
<65 years	2.64 (2.06–3.38)	
**Type of stroke**	
Ischaemic	–	**<0.001**
Intracerebral haemorrhagic	2.76 (1.85–4.12)	
Subarachnoid haemorrhagic	2.84 (2.17–3.71)	
**Hospital stay, weeks**	1.11 (1.05–1.17)	**<0.001**

*Concordance = 0.71. Likelihood ratio test = 179 on 5 df*.

*p ≤ 0.001*.

### Antiseizure Medications

**Figure 1:**
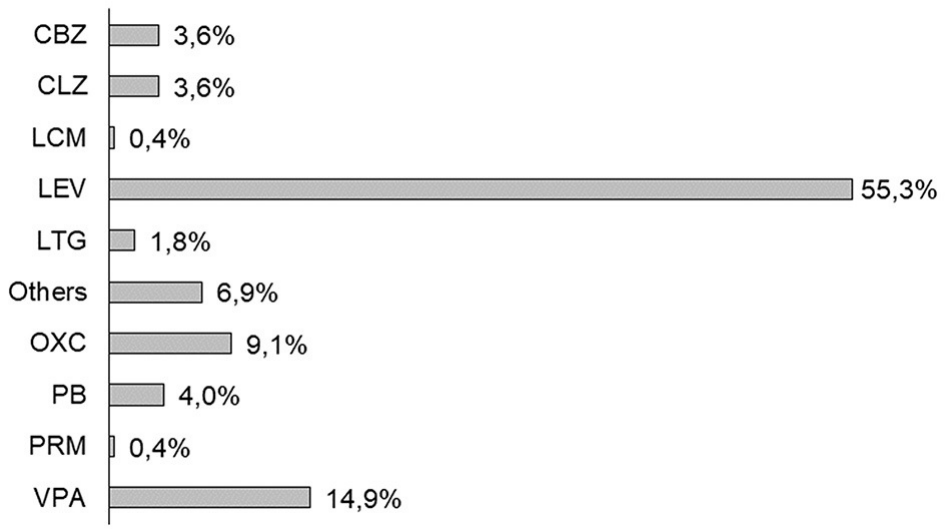
First antiseizure medication (ASM) prescription in post-stroke epilepsy (PSE) cohort (2013–2019). CBZ, carbamazepine; CLZ, clonazepam; LCM, lacosamide; LEV, levetiracetam; LTG, lamotrigine; OXC, oxcarbazepine; PB, phenobarbital; PRM, primidone; and VPA, valproate.

Monotherapy of ASM was prescribed in the 93% of patients with PSE (Figure 1). LEV was the most prescribed ASM (*n* = 152; 55.3%) for the management of PSE, followed by VPA (*n* = 41; 14.9%), and OXC (*n* = 25; 9.1%). In total, 20 patients with PSE needed more than one ASM already at the time of epilepsy diagnosis.

**Figure 2:**
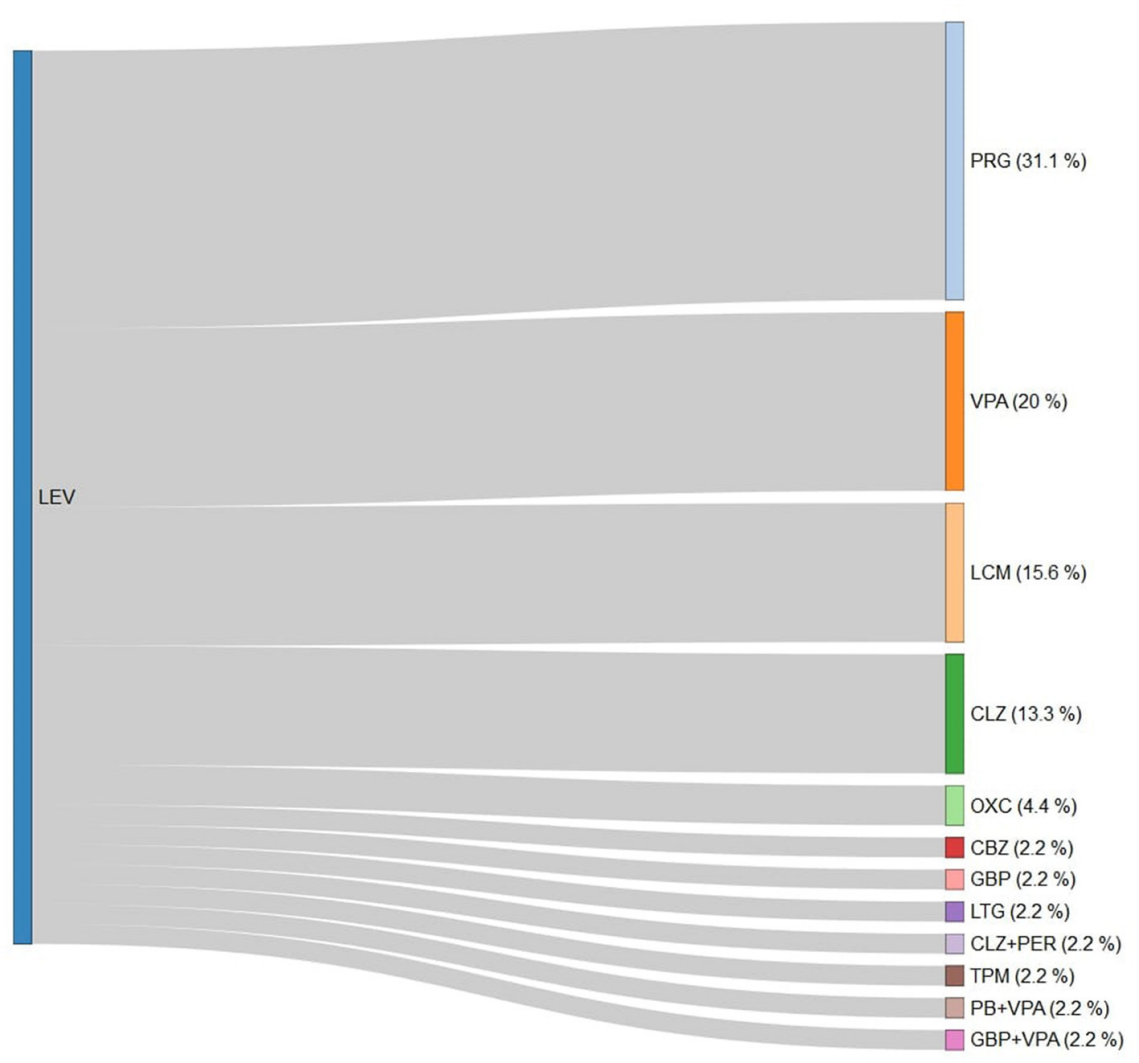
Antiseizure medication switch from levetiracetam in the study cohort. LEV, levetiracetam; PRG, pregabalin; VPA, valproate; LCM, lacosamide; CLZ, clonazepam; OXC, oxcarbazepine; CBZ, carbamazepine; GBP, gabapentin; LTG, lamotrigine; PER, perampanel; TPM, topiramate; and PB, phenobarbital.

Over follow-up, 44.7% (*n* = 123) of patients modified ASM with add-on or switch. About 12% of patients with PSE needed politherapy at last follow-up. LEV therapy was continued in the 63.2% of cases, and discontinued in 29.6% of cases (31% PRG, 20% VPA, and 16% LCM) (Figure 2). The second most prescribed ASM was VPA, which had a retention rate of 66%, and was discontinued in 24% of cases (30% PRG, 30% LEV, and 10% CBZ) (Supplementary Figure 1). The third ASM prescribed was OXC, which was switched in 40% of patients (40% CLZ, 30% LEV, and 20% PRG) (Supplementary Figure 2).

## Discussion

This regional study based on administrative healthcare data highlights that PSE develops in 3.1% of people with stroke within 4 years from the event. The risk of developing PSE seems particularly high after hemorrhagic events, such as SAH and intracerebral hemorrhage, and in younger people. Several studies have reported incidence and/or prevalence of PSE, with estimates ranging widely from 1 to 14% (14, 15) in relation to differences in cohort selection and ascertainment. Our estimates are in line with those provided by the largest national-level study based on health insurance database (*n* = 270,262), where late seizures were reported in 2.2% of people with stroke (16). Such finding lends weight to the reproducibility and accuracy of the catchment algorithm, such as the combination of administrative coding and ASM prescription (12, 16). Slightly higher prevalence was reported in survey-based (17) and prospective studies (18), as well as in studies focusing on seizures after hemorrhagic stroke (15). Despite such fluctuations in estimates, PSE happens in a consistent proportion of stroke survivors, and seems therefore worth of further investigations. This seems particularly true for hemorrhagic stroke, which, as in previous studies (15, 16, 19, 20), emerged as a consistent risk factor for developing PSE. The reasons underlying such peculiar susceptibility might reside in both direct and indirect mechanisms. Large brain hemorrhage can increase the risk of early and late seizures (3, 21), with early seizures favored by the loss of neurovascular unit integrity, disruption of the blood-brain barrier, and neurotransmitter release (3, 22). Epileptogenesis might then be supported by local gliosis, inflammation, and synaptic sprouting, with hemosiderin deposits able to induce a consistent increase in neuronal excitability (22–24). Other factors, beyond stroke subtype, emerge as critical predictors of PSE risk, such as younger age and length of hospital stay. The former is in line with previous reports on PSE after ischemic as well as hemorrhagic events (5, 18), and is included as an item in the CAVE score, a tool to calculate the risk of PSE after brain hemorrhage (21). Our data could not confirm the negative impact of stroke severity on PSE risk, although, as length of hospital stay could be a surrogate measure of injury severity, our results seem in line with available scores (2, 21). However, we must acknowledge that the length of hospital stay could be directly linked to the onset of seizures. Early seizures can increase the length of hospital stay and might partially contribute to the longer stay in people developing PSE, as they are also a risk factor for PSE. However, the length of hospital stay is mostly related to the severity of the stroke. Therefore, our data argue in favor of a higher risk of PSE in people with more severe stroke, which might in turn also have a higher risk of early seizures.

Despite being addressed by several studies about the risk factors and epidemiology of PSE, there is very limited evidence regarding the treatment choice and duration (25). Randomized controlled trials reported a consistent risk of drop-out from treatment, with 3–31% of people withdrawing from first ASM for adverse events (26, 27). Drop-out from treatment is frequently reported in prospective studies (20, 28), and might impact the seizure control and hospitalization (20). To this extent, early identification of adverse events through standardized scales and treatment switch and tailoring seems paramount (29). To date, data on treatment switch over time in PSE are lacking (30). In our study, LEV was the most prescribed ASM (55% of cases), followed by VPA and OXC. The 2013 International League Against Epilepsy (ILAE) report suggested GBP and LTG are established, CBZ is possibly, and TPM and VPA are potentially effective as initial monotherapy for elderly adults with newly diagnosed or untreated focal-onset seizures (31). European Stroke Organization (ESO) guidelines report data on CBZ, LEV, and LTG use in PSE, although the low power of available randomized trials prevented from drawing conclusions on optimal treatment. To date, LEV is considered the first choice in PSE because of the lower rate of recurrent seizures and fewer side effects (25). Tolerability profiles and drug-to-drug interactions remain crucial in choosing—or switching to/from—ASM in PSE. In our study, almost 30% of people started on LEV and VPA was switched to another ASM, a percentage that reached 40% with OXC. Several factors might have contributed to treatment switch. In a multicenter randomized open-label trial, SANAD II, LEV was significantly more likely to fail than LTG, and this treatment failure was due frequently to psychiatric adverse effects (32).

Moreover, the 2018 and 2021 European Society of Cardiology (ESC) guidelines provided strong restrictions on the concomitant use of direct oral anticoagulants (DOACs) and some ASMs (33, 34). Warning on the concomitant use of LEV with DOACs were issued, as DOAC levels can vary with coadministration of LEV, prompting potential switch to other ASMs (35, 36). Although paucity of data prevents our full understanding of DOAC-to-ASM interactions, which have been largely assumed on experimental only studies, our data seem in line with a trend toward shifting to ASM with fewer interactions over time, such as PRG or LCS. PRG, mentioned in the ESC guidelines as having no relevant interaction with DOACs (33), might be preferred to LEV to impact on neuropathic pain and mood disturbances, which are indeed frequent with the former (37). On the other side, the choice of switching to LCM might reside in easy administration and schedule, no interaction with DOAC, and potential neuroprotective effects suggested from experimental studies (38). The rate of switch to LCM should be interpreted in the light of late marketing in Italy, which approved LCM monotherapy only in very late 2018. Therefore, switch rates might have been underestimated, with a substantial proportion of people with PSE expected to have a first or second prescription of LCM in more recent times, according to the favorable efficacy and tolerability profile (39).

### Limitations and Strengths of the Study

Our study has limitations and strengths intrinsic to healthcare regional database studies. Large registry-based studies serve as a useful complement to smaller studies, which are often subject to more inclusion bias but have more detailed information counting on individual medical records. The use of administrative date may collect large amount of data, but lacks some information, such as stroke severity, volume, or location of lesion or hemorrhage, or subtle adverse events from medications. Nevertheless, the large amount of data collected allow to refine our management on a global scale, and in a specific patient subgroup given the accuracy of the catchment method. The algorithm is used for a single Italian Region with a 900,000 people. Future study will collect data from more regions to cross-validate these findings. A second limitation stands in the outcomes we selected. However, as clinical data are lacking, investigating stroke recurrence or seizure recurrence over time could have been biased by the functional status of the patient after the first event. Therefore, limiting the analysis to prescription patterns has ensured an overall follow-up on medications, allowing a preliminary interpretation of treatment switch.

## Conclusion

Our study, counting on regional healthcare data, highlighted that PSE can happen in up to 3.1% of people after stroke. Hemorrhagic events and younger age carry a higher risk of developing PSE. First ASM is switched in 30% of cases: therefore, first choice should address potential drug-to-drug interaction and tolerability that can limit the compliance or impact on secondary prevention.

## Data Availability Statement

The raw data supporting the conclusions of this article will be made available by the authors, without undue reservation.

## Ethics Statement

The Regional Umbria ethical review board approved the study (No. 3420/19). Written informed consent for participation was not required for this study in accordance with the national legislation and the institutional requirements.

## Author Contributions

CCo, EN, and MR participated in study design, analyzed data, and wrote first, and final draft. MR, DF, and PE performed statistical analysis. CCo, EN, PE, DF, PCas, MD, CCa, and MR collected data. LP and PCal revised the manuscript for intellectual content. All authors contributed to the article and approved the submitted version.

## Funding

This research project has been in part supported by unconditional contribution of UCB Pharma. UCB Pharma did not have any role in study design, in the collection, analysis, and interpretation of data, in the writing of the manuscript, and in the decision to submit the article for publication.

## Conflict of Interest

PCal receives research support from Preston and Zambon to perform preclinical investigation on drugs that are not discussed in the text. The remaining authors declare that the research was conducted in the absence of any commercial or financial relationships that could be construed as a potential conflict of interest.

## Publisher's Note

All claims expressed in this article are solely those of the authors and do not necessarily represent those of their affiliated organizations, or those of the publisher, the editors and the reviewers. Any product that may be evaluated in this article, or claim that may be made by its manufacturer, is not guaranteed or endorsed by the publisher.
