# Supplementary material for: Incidence and Antiseizure Medications of Post-stroke Epilepsy in Umbria: A Population-Based Study Using Healthcare Administrative Databases
Source: Front Neurol. 2022 Jan 12;12:800524. doi: 10.3389/fneur.2021.800524 (PMC8790124; doi:10.3389/fneur.2021.800524)
Supplement: Supplementary file 1 [file Data_Sheet_1.PDF]

Supplementary material – Figure I. Switch from valproic acid in the study cohort.

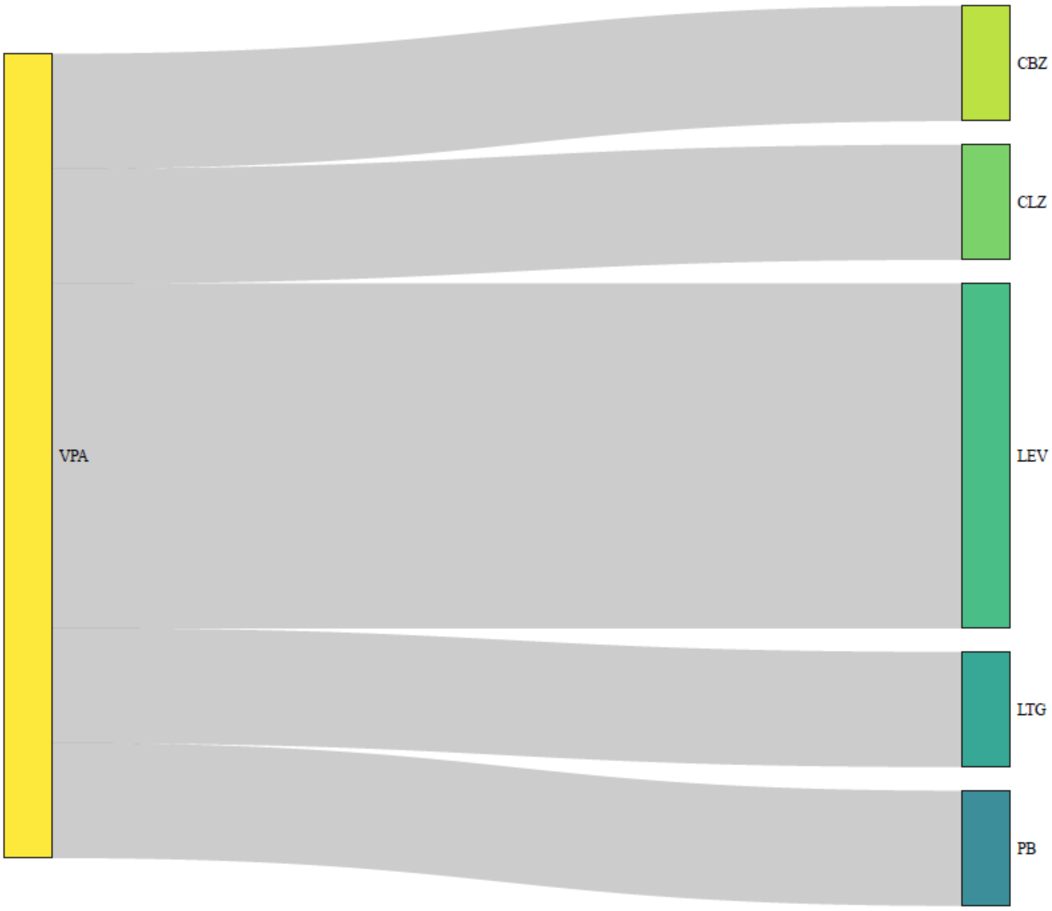

Legend: valproate (VPA), carbamazepine (CBZ), clonazepam (CLZ), levetiracetam (LEV), lamotrigine (LTG), phenobarbital (PB).

Supplementary material – Figure II. Switch from oxcarbazepine in the study cohort.

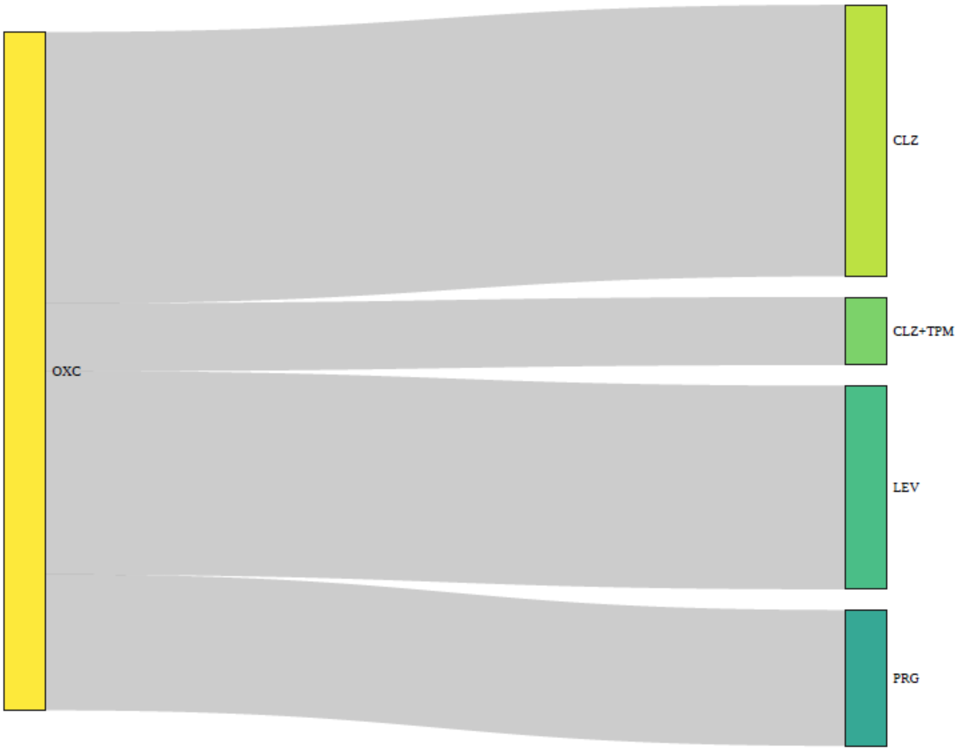

Legend: oxcarbazepine (OXC), clonazepam (CLZ), topiramate (TPM), levetiracetam (LEV), pregabalin (PRG).
